# Novel Biomarkers Aim at Detecting Metastatic Sentinel Lymph Nodes in Breast Cancer

**DOI:** 10.29252/ibj.24.3.183

**Published:** 2020-01-26

**Authors:** Behnaz Bakaeean, Mehran Gholamin, Seyed Abbas Tabatabaee Yazdi, Mohammad Naser Forghani

**Affiliations:** 1 Department of Biology, Marvdasht Branch, Islamic Azad University, Marvdasht, Iran;; 2 Department of Biology, Science and Research Branch, Islamic Azad University, Marvdasht, Iran;; 3 Department of Laboratory Sciences, School of Paramedical Sciences, Mashhad University of Medical Sciences, Mashhad, Iran;; 4 Department of Pathology, Mashhad University of Medical Sciences, Mashhad, Iran;; 5 Surgical Oncology Research Center, Mashhad University of Medical Sciences, Mashhad, Iran

**Keywords:** CLEC3A, Kallikreins, Sentinel lymph node

## Abstract

**Background:**

Intra-operative molecular diagnostic assays are currently used for the detection of lymph node metastases. The objective of this study was to find new biomarkers to improve diagnostic accuracy in the detection of metastatic axillary lymph nodes in breast cancer patients.

**Methods:**

We applied an absolute quantitative real-time RT-PCR to quantitate the expression of *CK19*, *KLK11*, and *CLEC3A* mRNAs in 79 FFPE SLNs from 35 breast cancer patients. The* CK19* was confirmed as a standard biomarker, and the level of expression of selected new markers, *KLK11* and *CLEC3A*, was evaluated in pathologically negative and positive SLNs by using absolute quantitative real-time PCR.

**Results:**

The overall concordance of the *CK19* gene with pathological results was 92.4% (less than 250 copies) in negative SLNs and 85% in positive SLNs (more than 250 copies). The sensitivity and specificity of *CK19*, which were detected by real-time PCR, was 85% and 46%, respectively. Our results revealed that lower *CLEC3A* was associated with more lymph node involvement. We could set a cut-off point for *CLEC3A* with the sensitivity of 78% and specificity of 60%. Also, the mean *KLK11* had a statistically significant reverse correlation with tumor grade (*p* = 0.017). Higher *CK19 *levels were related to more tumor invasion (*p* < 0.0001).

**Conclusion:**

Regarding the findings, *CLEC3A* along with *CK19* can be used as a promising marker with high sensitivity and specificity for the detection of metastatic SLN*. *

## INTRODUCTION

Breast cancer is the common cause of death amongst women and the leading cause of morbidity and mortality worldwide^[^^1^^]^. Axillary lymph nodes status has a vital role in determining the survival status of patients and prognosis of the disease, helping clinicians to decide the most appropriate surgical procedure and subsequent treatment options^[^^2^^]^. 

A SLN is the first lymph node or groups of nodes to which cancer cells are probably spread from a primary tumor. SLN biopsy is a surgical procedure to diagnose if cancer has spread to the lymphatic system^[^^3^^]^. Frozen section or touch imprint is a routine method used during surgery for the analysis of the SLN and allow rapid H&E staining^[^^4^^]^. Understandably, the accuracy and dependability of these methods rely heavily on the expertise of a cytopathologist and may vary based on institution or clinical setting^[^^5^^]^. Due to mistakes in common pathological techniques and the false-negative results, a sensitive and simple method for accurately determining the staging of breast cancer is needed^[^^7^^]^. RT-PCR is a highly sensitive diagnostic tool able to detect molecular biomarkers such as *MUC1*, *CK19*, and carcino-embryonic antigen in lymph node metastases in patients with invasive breast cancer^[^^8^^-^^10^^]^. 

*CK19* is one of the most popular molecular biomarkers and an epithelial cell marker that is not expressed in normal axillary lymph node tissue^[^^11^^]^. The amount of *CK19* mRNA expression is related to the level of metastatic foci^[^^12^^]^. *CK19* mRNA, the most proper marker, exists in high levels in metastatic (not non-metastatic) lymph nodes. Accordingly, it has a high sensitivity potency and ability to identify metastatic from non-metastatic lymph nodes^[^^11^^]^. Based on previous research, the cut-off value is determined by the number of copies of *CK19* mRNA as a criterion to distinguish negative nodes (less than 250 *CK19* mRNA copies) from micrometastases (250–5000 *CK19* mRNA copies or >0.2–2 mm in diameter) and macrometastases (more than 5000 *CK19* mRNA copies)^[^^13^^]^. 

According to the recent next generation sequencing studies, *CLEC3A* and *KLK11*, are overexpressed in metastatic lymph nodes^[^^14^^]^. *CLEC3A* is a protein related to the great family of C-type lectins and can be seen in normal human breast tissue, but not in any other normal human tissue^[^^15^^-^^17^^]^. This protein is a heparin-binding, cell adhesion modulator that have capability to alter tumor cell invasion and metastasis by modulating tumor cell adhesion and the plasminogen/plasminogen-activator system^[^^18^^]^. Kallikreins are a subgroup of serine proteases, enzymes capable of cleaving peptide bonds in proteins, and a family of 15 genes on chromosome 19^[^^19^^]^. Studies have revealed the expression of *KLK11* in ovarian, prostate, breast, lung, pancreas, and colon cancer tissue^[^^20^^-^^22^^]^. It has also been indicated that *KLK11* expression is regulated by steroid hormones such as estrogen^[^^23^^]^. *CLEC3A* and *KLK11* are not *normally* expressed in *lymph node* tissue^[^^14^^]^. Furthermore, there are limited investigations on the presence of this biomarker in metastatic lymph nodes in breast cancer. The aim of this study was to find new biomarkers to improve the diagnostic accuracy in the detection of metastatic axillary lymph nodes. We used *CK19* expression as a standard diagnostic tool.

## MATERIALS AND METHODS

**Patients and source of SLN**

SLNs (n = 78) were obtained from axillary lymph node dissection. The specimens of 35 breast cancer patients in clinical stages I and II were acquired from the Pathology Department of Pastorno Hospital, Mashhad, Iran. All patients had operations by the same surgical team and had received no chemotherapy from February to December 2017. Based on the permanent section H&E analysis of SLNs, the specimens were divided into two groups of reactive and metastatic lymph nodes.

**RNA extraction**

Five to six histological sections, 5 µm in thickness, were cut from each FFPE block. Afterwards, deparaffinization was carried out using xylene, according to the Qiagen (Valencia, CA, USA) protocol. Paraffin was first dissolved by adding 1 ml of xylene and then centrifuged at full speed for 2 minutes. The supernatant was carefully removed, and then 1 ml of ethanol (96-100%) was added to the pellet and mixed by vortexing and centrifuged at full speed for 2 minutes. The supernatant was removed carefully by pipetting then the pellet was dried at room temperature. Next, 240 µl of buffer PKD and 10 µl of proteinase K were added, respectively, mixed by vortexing, and incubated at 56 °C for 15 min, and finally at 80 °C for 15 min. RNA was purified by RNeasy FFPE Kit (Qiagen, Valencia, CA), and the RNA quality was confirmed by gel electrophoresis. 

**cDNA synthesis**

In reverse transcription reactions, cDNA was synthesized using the PrimeScript^TM^ RT Reagent Kit (TaKaRa, Japan) in accordance with the manufacturer’s protocol (37 °C for 15 min and 85 °C for 5 s). The cDNA quality was confirmed by the amplification of glyceraldehyde-3-phosphate dehydro-genase as a control.

**Construction of standard curves for the** ***CK19, CLEC3A*** **, and** ***KLK11*** **copy number determination**

Specific controls were constructed for *CK19*, *CLEC3A*, and *KLK11* by TA cloning of PCR products. Plasmid pBlusScript SK II (+) was used to clone the desired fragment. The recombinant vector was transformed into competent *E. coli *DH5-α, and the transformed culture was spread on agar Luria-Bertani plates containing ampicillin (100 µg/ ml), IPTG (0.1 mM), and X-gal (20 μg/ml) and incubated at 37 °C for one night. Transformed (white) colonies were picked and processed for plasmid isolation. 

**Fig. 1:**
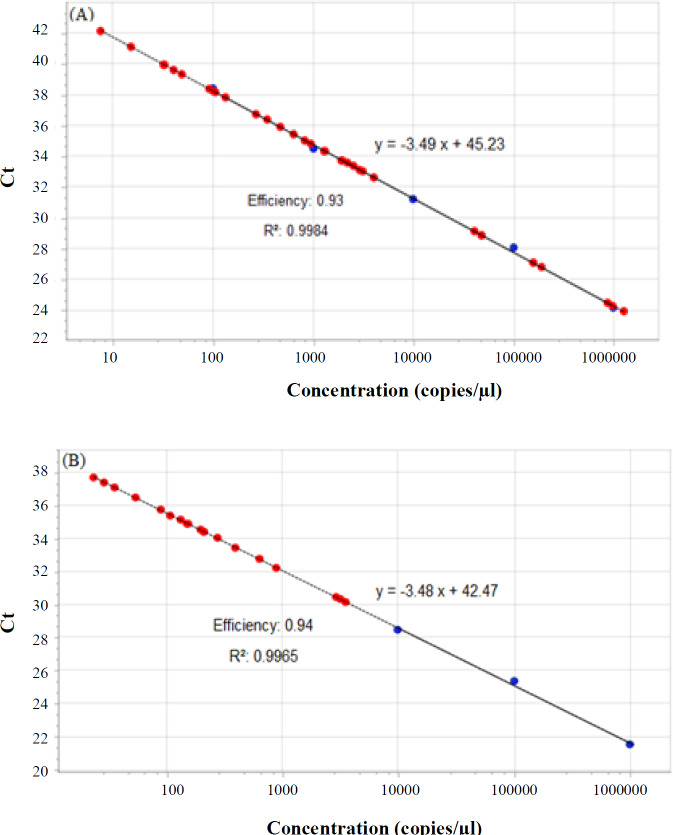
Standard curves for (A) *CK19* and (B) *CLEC3A*. The Ct is shown on the Y axis, and standard serial dilutions from 10^6^ to 10^2^ (copies/μl) is indicated on the X axis. The correlation coefficient (R^2^) of *CK19* and *CLEC3A* were 0.93 and 0.94, respectively. Blue and red circles show standards and samples, respectively

Plasmid purification was carried out using mini-prep protocol. To prepare standards with known concentrations for a standard curve, the molar concentration of the extracted plasmid was measured with NanoDrop, and then the dilution was made. Finally, standards with a concentration range of 10^6^ to 10^2^ were used to draw the standard curve (Fig. 1). For all the standards, copy numbers were calculated as below^[^^24^^]^:

DNA copy=6.02×1023copies mol-1× DNA amount (g)DNA lenght bp× 660 (g mol-1 bp-1)

Absolute quantitation demonstrates the precise copy concentration of the target gene, but relative quantification determines fold changes in the expression between two samples. Absolute quantitation uses well-known diluted serial standards, and then the standard curve is designed. The standard curve provides a linear relationship between Ct and the initial values of the entire RNA or cDNA, which allows determining the unknown concentration based on its Ct values. 

**Real-time PCR assay**

The TaqMan® real-time PCR method was performed by using StepOne™ Real-Time PCR. Specific oligonucleotide primers and probes were designed and synthesized by *Macrogen, Korea* (Table 1). Thermal cycling conditions were designed as follows: initial denaturation at 95 ºC for 15 min, followed by 40–45 cycles at 95 º◦C for 30 s, 60 ºC for 30 s, and 72 ºC for 30 s. All reactions for each gene in reactive and positive samples were run in triplicate. 

**Statistical analysis**

 Statistical analysis was performed using SPSS22 software. Results were reported as mean ± standard deviation. The Kolmogorov–Smirnov test was used for normal or abnormal distribution of the data, as well as in percentage descriptions. The relationship between the expression of biomarkers and cancer histology was assessed by linear regression analysis (Mann***–***Whitney U test). Kruskal Wallis was employed to compare the groups. The ROC analysis and the AUC were calculated to evaluate the diagnostic values of the markers. Statistically significant correlation was indicated by *p* < 0.05.

**Ethical statement**

The above-mentioned sampling protocols were approved by the Ethics Committee of Mashhad University of Medical Sciences (ethical code: IR. mums.fm.rec. 1396.265). Written informed consents were provided by all the participants. 

**Table 1:** Sequences of primers and probes used in real-time RT-PCR

**Primer/probe**	**Oligonucleotide sequence (5'-3')**
CK19 F	5'-GGC CTA CCT GAA GAA GAA CCA-3' (21 mer)
CK19 R	5'-AAT CCA CCT CCA CAC TGA CC-3' (20 mer)
CK19 probe	5'-FAM-AGT ACG CTG AGG GGC CAA G-BHQ1-3' (19 mer)
	
KLK11 R	5'-GAT GGT GAT GTT GGC GCA T-3' (19 mer)
KLK11 F	5'-CAG CTG CCT CAT TTC CGG-3' (18 mer)
KLK11 probe	5'-FAM-CAG TTA CGC CTG CCT CAC AC-BHQ1-3' (20 mer)
	
CLEC3A F	5'-GGA CTT GTA ATT TGC ATC CTG GT-3' (23 mer)
CLEC3A R	5'-CCA GAG CTT TTC AAT TTG AGT CT-3' (23 mer)
CLEC3A probe	5'-FAM-CAG GAA GCA CAG CAA ACG TC-BHQ1-3' (20 mer)

F, forward primer R, reverse primer

**Table 2:** Clinicopathological characteristics of patients

**Characteristics**	**Patients (n = 35)**
Age (y)	
Total ≤ 50 > 50 Mean	35 15 20 50/2
Histological type	
Ductal Lobular	34 1
Grading	
GI GII GIII	5 22 8
Tumor size	
T1 (0-1.9) T2 (2-3.9)	15 20
Tumor stage	
IA IIA	15 20
Clinical lymph node status	
N1 N2	30 5
Pathologic stage	
IIA IIB IIIA	14 16 5
Estrogen receptor	
Negative Positive	8 27
Progesterone receptor	
Negative Positive	9 25
Her2/neu	
Negative Positive	26 9
Ki67	
Negative Positive	3 32

## RESULTS

**Patient histopathological characteristics**

A total of 79 lymph nodes from FFPE samples of 35 breast cancer patients were evaluated. It should be noted that patients did not receive any neoadjuvant therapy. According to the pathology results, we stratified patients on the basis of their pathologic status of SLNs into two groups. The first group (I) consisted of 15 patients with 27 pathologically negative lymph nodes, and the second group (II) included 20 patients with 52 metastatic lymph nodes. The age of the patients ranged from 27 to 68 years (mean 50.2), and the clinicopathological findings of these patients are depicted in (Table 2).

**Quantitation of** ***CK19, KLK11*** **, and** ***CLEC3A*** **mRNAs expression in lymph nodes**

 We used absolute quantitative real-time RT-PCR to determine the expression of *CK19*, *KLK11*, and *CLEC3A* mRNAs, pathologically negative and positive lymph nodes. The actual copy numbers of target genes were also determined by relating the Ct value to a standard curve. The expression levels of the three mRNAs differed histopathologically between positive and negative lymph nodes (Table 3).

**Expression analysis of** ***CK19*** **,** ***KLK11*** **and** ***CLEC3A*** **mRNAs in samples **

***CK19 gene expression***

**Fig. 2.**
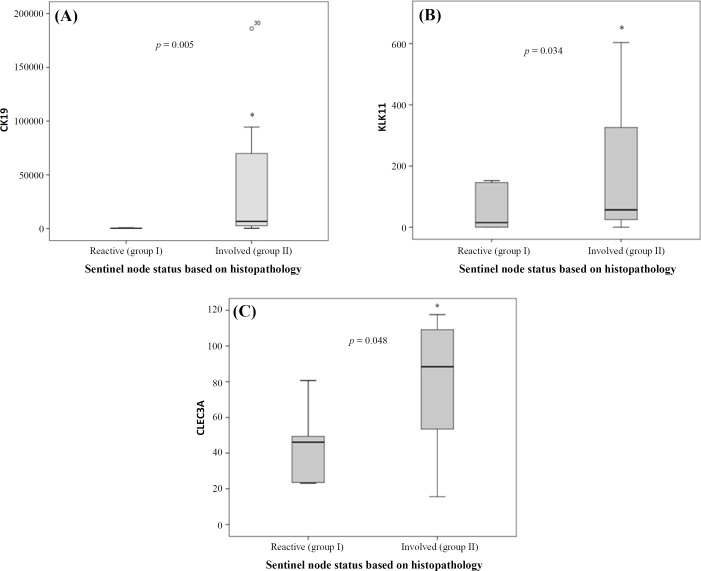
Comparing mRNA expression in group I (reactive) and group II (involved) of lymph nodes. (A) *CK19*, (B) *KLK11*, and (C) *CLEC3A* expressions. ^*^The expression of all markers in group II significantly increased compared with group I. Lines in the middle show the mean expression value

**Fig. 3:**
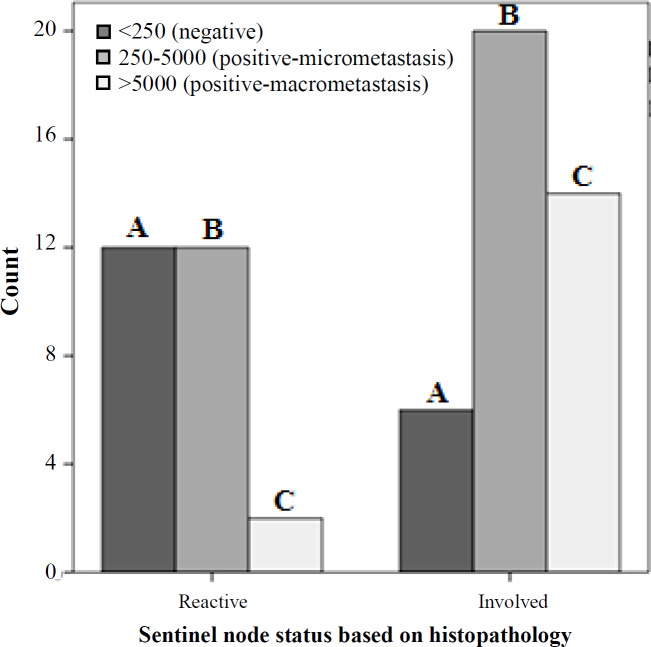
Correlation between histopathology results and *CK19* cut-off number in the entire series of 79 SLNs. Group I (reactive): by *CK19* copy number (A) 46.2% negative, (B) 46.2% micrometastasis, and (C) 7.7% macrometastasis. Group II (involved): by *CK19* copy number (A) 15% negative, (B) 50% micrometastasis, and (C) 35% macrometastasis, (*p *= 0.005).

Based on the real-time PCR data, the minimum and maximum values of *CK19* expression among all samples were 15.1 and 1028629.6 copies/µL, respectively, and a statistically significant (*p* = 0.005) up-regulation of *CK19 *was found in group II compared to group I (Fig. 2A). Expression of the *CK19* mRNA, according to cut-off numbers, are illustrated in Figure 3. As shown in group I (reactive), the results were as 46.2% negative, 46.2% micrometastasis, and 7.7% macrometastasis (false-negative cases). Group II (involved) demonstrated 50% micrometastasis and 35% macrometastasis, which is expected and consistent with pathologic reports. Based on the Mann-Whitney U test, the mean *CK19* between the two groups was statistically significant (*p *= 0.005).

**Table 3:** Mean Ct values and mean copy numbers of the *CK19*, *KLK11*, and *CLEC3A* using real-time RT-PCR

**Mrn** **A markers**	**Nodal status**	**Mean** **Ct value**	**Mean copy number**
*CK19*	Pathologically reactive node (group I)	37.57	374.20
	Pathologically involved node (group II)	32.97	48351.90
			
*KLK11*	Pathologically reactive node (group I)	37.02	62.96
	Pathologically involved node (group II)	35.06	194.70
			
*CLEC3A*	Pathologically reactive node (group I)	35.60	44.50
	Pathologically involved node (group II)	34.76	78.10

***KLK11 gene expression***

The average value of *KLK11* mRNA expression for groups I and II were 62 and 194 copies/µl, respectively (Fig. 2B), and the lowest limit of detection was 0.1386. Based on the Mann-Whitney U test, the mean *KLK11* expression difference between the two groups was not statistically significant (*p = *0.034).

***CLEC3A gene expression***

The expression levels of *CLEC3A* mRNA in groups I and II were 44 and 79 copies/µL, respectively (Fig. 2C). The minimum limit of detection was 15.581 copies/µL. Based on Mann-Whitney U, a statistically significant (*p* = 0.048) up-regulation of *CLEC3A* mRNA was observed between the two groups. Also, there was an independent correlation among the three markers (*p* > 0.05). 

**ROC** **curve analysis**

**Fig. 4:**
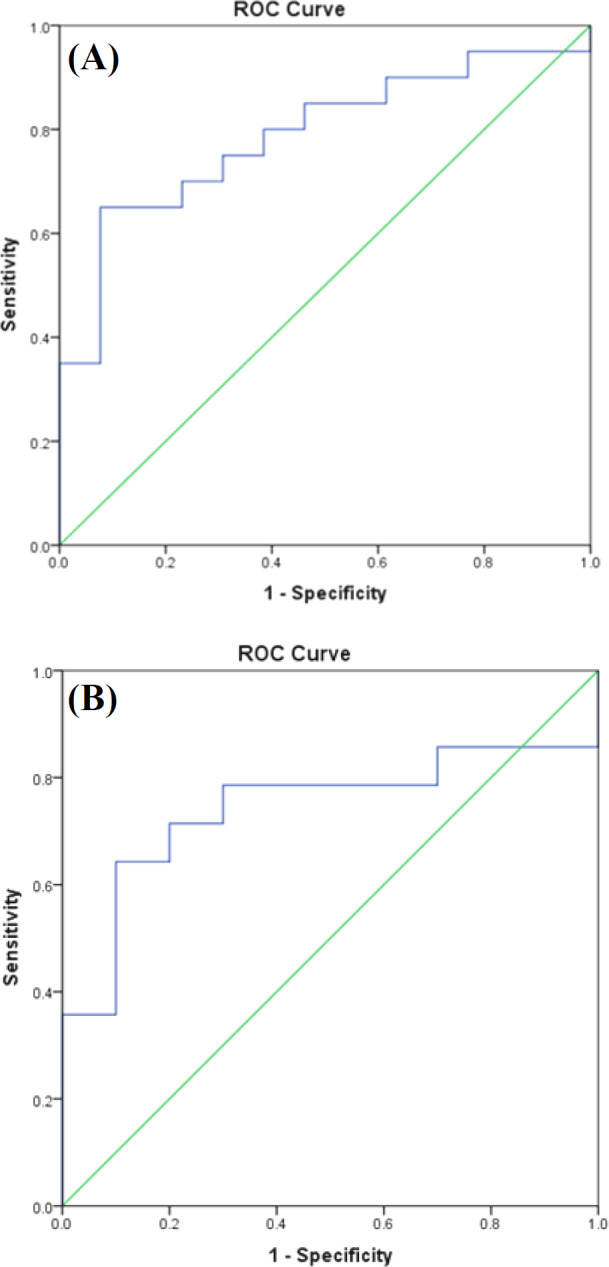
ROC curve for (A) *CK19 (*AUC = 0.788) and (B) *CLEC3A (*AUC = 0.743).

The diagnostic value of the *CK19*,* KLK11*, and *CLEC3A *mRNAs was quantified by the ROC curve (Fig. 4). This discrimination was measured by the AUC. The AUC for CK19 (*p* = 0.006) indicated that the results of the ROC analysis were reliable. The AUC = 0.788 (95% CI: 0.688-0.945) was consistent with the moderate accuracy test. According to the 250 copies cut-off point for this gene, the specificity was 46%, and the sensitivity was 85%.

The AUC for *KLK11 *was not meaningful (*p* = 0.310). In *CLEC3A*, the AUC was significant (*p* = 0.046); therefore, the results showed to be reliable. The amount of AUC = 0.743 (95% CI: 0.535-0.951) corresponded to the moderate test accuracy. According to the ROC analysis, we identified a cut-off of 50 copies/µL *CLEC3A* mRNA with 78% sensitivity and 60% specificity. Depending on the determined cut-off for the *CLEC3A,* in group I, patients were 60% *CLEC3A*-negative and 40% *CLEC3A*-positive, and in group II, 21.2% *CLEC3A*-negative and 78.5% *CLEC3A*-positive. The percentage of positive and negative SLNs on the basis of the cut-off number of *CLEC3A* and CK19 is presented in Table 4.

**Fig. 5:**
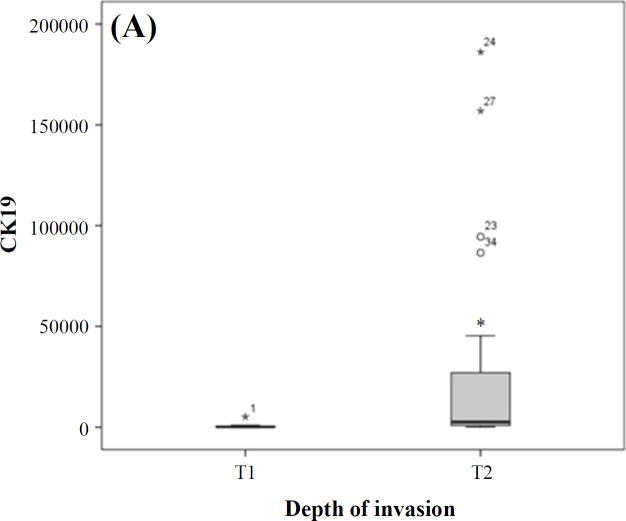
(A) Relationship between *CK19* and tumor invasion depth (T). ^*^CK19 expression in invasion depth (T2) is higher than (T1) (*p* < 0.0001); (B) relationship between *KLK11* and tumor grades. ^*^KLK11 expression in grade I is higher than grade II and III (*p* = 0.017)

The evaluation of the relationship between *KLK11* expression and patients with different nuclear grades by using the Kruskal-Wallis statistical test indicated that the average *KLK11* with tumor grade was statistically significant (*p* = 0.017), and in grade I, it was higher than grade II and III (Fig. 5B). It seemed that the lower levels of *CLEC3A* were associated with the greater involvement of the lymph node (N2 versus N1).

The mean *CK19 *had a statistically significant association with tumor invasion depth (T; Mann-Whitney U test, *p* < 0.0001; Fig. 5A), but this relationship was not significant for the other two markers (*p* > 0.05). A lower value of *CK19* was associated with a lower invasion depth (T1 versus T2).

The mean *CK19* was statistically correlated with the stage of the tumor (Kruskal-Wallis test, *p* = 0.001), but this relation was not significant for the other two markers (*p* > 0.05). It seemed that the amount of *CK19* at the lowest stage (IIA) was less than the two other stages. 

## DISCUSSION

A variety of reports have described more accurate diagnosis of micrometastasis in axillary lymph nodes, by using reverse transcription of some indicators such as prolactin-induced protein, *CK19*, mammaglobin, carcinoembryonic acid, and *MUC1*. Among these makers, *CK19* and mammaglobin have illustrated high sensitivity and specificity for the detection of lymph node metastasis of breast cancer^[^^25^^]^. Due to high reliability, these two markers have currently used clinically.

*CK19* is known as an epithelial cell marker and is widely expressed in more than 90% of breast cancers. In previous studies of *CK19* detection, one-step nucleic acid amplification was identified as a valuable intra- operative approach for the diagnosis of lymph node metastases in patients with breast cancer and defined as having the highest sensitivity (about 90%)^[^^26^^,^^27^^]^. Based on *CK19* cut-off numbers, we observed 92.4% negative nodes (less than 250 copies) for group I and 85% positive lymph nodes (more than 250 copies) for group II, which can be expected and is consistent with the pathologic results. Fujisue *et al.*^[^^28^^]^ have reported that the negative cases of *CK19* are clearly associated with a negative level of ER-PR and higher levels of *Ki67* expression, as well as higher nuclear grade and higher expression of P53. In cases where breast cancer is triple-negative, the expression of *CK19 *is lower. On the contrary, we observed no significant relationship between chosen biomarkers and ER-PR and *Ki67* and Her2/neu expression. Although extensive research has been carried out on identified markers for detecting lymph node involvement in breast cancer, there is still a need to identify newer markers with higher sensitivity and specificity.

**Table 4:** Sensitivity and specificity of real-time RT-PCR of *CK19* and *CLEC3A*

***CLEC3A *** **(cut-off value = 50)**		***CK19 *** **(cut-off value = 250)**			**Nodal status**
**Negative (%)**	**Positive (%)**	**Negative** **<250 (%)**	**Micrometastasis** **250-5000 (%)**	**Macrometastasis** **>5000 (%)**	
46.2	46.2	7.7	60	40	Pathologically reactive node (group I)
15.0	50.0	35.0	21.2	78.5	Pathologically involved node (group II)
0.85		0.78			Sensitivity (%)
0.46		0.60			Specificity (%)

Pursuant to the next-generation RNA sequencing study by Feng Liang *et al.*^[^^14^^]^ in non-SLN-positive group, *CYP2A13*, *KLK11*, and *CLEC3A* demonstrated higher overexpression. Biomarkers identified in this study can provide a new understanding of the mechanism of breast and lymph node involvement, as well as the selection of patients for surgery. Hence, we selected *KLK11* and *CLEC3A* as new biomarkers to detect metastatic SLNs. Evidence has suggested that Kallikreins play a role in cancer, and some of them are potentially new markers of cancer and other biological diseases^[^^29^^]^. It has also been shown that the expression of *KLK11* in breast cancer contributes significantly to the progression of cancer by increasing the bioavailability of IGF through degradation of IGFBP-3^[^^30^^]^, and extremely significant expression of *KLK11* was observed in patients with breast cancer grades I and II compared to III. In agreement with the results reported by Sano *et al.*^[^^30^^]^, our results displayed a significant reverse correlation between overexpression of *KLK11* and tumor grade. *CLEC3A* is specifically expressed in the cartilage, and a significant expression in the breast and colon cancer tissue has been identified^[^^16^^,^^31^^]^. The expression of *CLEC3A* in breast IDC was higher than the normal tissue of the breast and axillary lymph nodes (pathologic N1 versus N0). Increasing the expression of *CLEC3A* may correlate with the metastatic potential of the IDC breast, which indicates a poor prognosis in the IDC of the breast^[^^32^^]^.

Based on our results, the mean expression of this gene was significantly different between the two groups. In order to obtain the highest sensitivity and specificity with an optimal cut-off value, the ROC curve analysis was utilized. ROC analysis indicated the absolute sum of sensitivity and specificity regarding the single copy number cut-offs. The best cut-off for our purpose was specified with a higher AUC. The cut-off value was estimated at 50/µL copies of *CLEC3A *mRNA, on the basis of the ROC analysis with an AUC equal to 0.743, copy number of 50/µL, indicating the sensitivity of 78% and a specificity of 60%. The results from the analysis of *CLEC3A* expression levels revealed a higher *CLEC3A *level in metastatic SLN compared to normal tissue (N0 versus N1) and also a higher expression in N1 versus N2 (*p* = 0.04). Positive and negative predictive values of this test were 100% and 35%, respectively, and the diagnostic value of the *CLEC3A* gene can be as much as *CK19*.

In summary, although very limited study has been conducted on the expression levels of *KLK11 *and *CLEC3A* mRNAs in the SLN tissue in breast cancer, we observed the overexpression of these genes in the positive SLN tissue similar to the *CK19* biomarker. Based on our results, the expression of all the three biomarkers increased in group II without any correlation among them. We also found a significant correlation between mean *KLK11* and *CLEC3A* values with nuclear grade (G) and lymph node status (N), respectively. Additionally, higher *CK19* values ​​were found to be associated with a more invasive tumor, involvement of the SLN, and a higher stage of cancer. We set a cut-off point for *CLEC3A*, but more precise cut-offs can be determined by increasing the number of patients and following up with them. The expression profile of *CLEC3A*, as a useful benchmark described in this study, supports the clinical utility of this biomarker in the diagnosis of metastatic SLNs in breast carcinoma, but more encouraging results merit further investigation.
